# Patterns of Prescription of Psychotropic Medications and Their Adherence among Patients with Schizophrenia in Two Psychiatric Hospitals in Accra, Ghana: A Cross-Sectional Survey

**DOI:** 10.1155/2018/9850594

**Published:** 2018-01-01

**Authors:** Sharon Ashong, Irene A. Kretchy, Barima Afrane, Ama de-Graft Aikins

**Affiliations:** ^1^Department of Pharmacy Practice and Clinical Pharmacy, School of Pharmacy, University of Ghana, Legon, Ghana; ^2^Regional Institute for Population Studies, University of Ghana, Legon, Ghana

## Abstract

**Background:**

Patients with schizophrenia are managed with antipsychotics and other psychotropic medications.

**Objectives:**

This study aimed to assess the commonly prescribed psychotropic medications for patients with schizophrenia, explore the types of therapeutic monitoring that were performed, and find out whether the side effects experienced by the patients played any role in their adherence behaviour.

**Methods:**

This hospital-based cross-sectional study enrolled 259 patients with schizophrenia from Accra Psychiatric Hospital and Pantang Psychiatric Hospital. Data were collected on mental status, side effects, types of therapeutic monitoring performed, and adherence behaviour.

**Results:**

Olanzapine was the commonly prescribed psychotropic medication. Most of respondents (73.4%) experienced mild levels of side effects. The negative effects were predominantly genitourinary (26%) and gastrointestinal (17.2%). Blood pressure and heart rate measures were the main types of monitoring performed but no measurement of drug levels was reported. About 98.1% of the participants poorly adhered to their medications and the major reasons for poor adherence were economic challenges, forgetfulness, and the feeling of wellness.

**Conclusion:**

Adherence to medication is a major health problem among patients with schizophrenia and there is a need to improve adherence and treatment outcomes.

## 1. Introduction

Mental disorders account for a growing proportion of the worldwide burden of diseases. In 2013, mental disorders accounted for 14% of the worldwide burden of diseases, a 2% rise in prevalence over a period of a decade [1]. In Ghana, the estimated prevalence of mental disorders is 13%; the prevalence of severe mental disorders like major depression and schizophrenia is estimated at 3% [2, 3]. The main form of treatment of severe mental disorders, especially among hospitalized patients, is the use of psychotropic medications. In Ghana, psychotropic medication treatment in the psychiatric hospital setting has a 100-year history, beginning in 1906 at the Accra Psychiatric Hospital (APH) [4, 5]. The use of medications is essential to improve the debilitating effects of mental disorders and to prevent associated complications [6].

Schizophrenia is a complex chronic mental health disorder characterized by an array of symptoms, including delusions, hallucinations, disorganized speech or behaviour, and impaired cognitive ability [7]. Schizophrenia is debilitating and persistent and is associated with poor physical health, early mortality, and substantial impairment in functioning [8]. While treatment includes pharmacotherapy and psychosocial interventions, antipsychotics are the cornerstone of pharmacological treatment for schizophrenia [9]. Patients with schizophrenia take psychotropic medications for an extended period, and for these patients, medication adherence is critical to maintain adequate symptom control and achieve better health outcomes [10]. Adherence is defined as the degree to which patients take medications as indicated by their practitioners [11], yet, this remains a major problem, with average rates of adherence to antipsychotic medications generally between 40 and 50% [12]. Nonadherence can lead to relapses, hospitalization of patients, and increase in death rate [13]. However, patients on long-term psychiatric treatment usually experience complex side effects such as gastrointestinal disturbances, weight gain, and sexual dysfunction [14, 15]. These side effects undermine their ability to take their medicines regularly. Poor adherence to therapy and the experience of side effects associated with psychotropic drug use have accounted for relapse among patients with schizophrenia [16–18].

About 75% to 90% of psychiatric patients cared for and discharged in some psychiatric hospitals are readmitted due to relapse [19] which may be attributed to poor medication adherence [20]. Numerous factors have been determined to affect patient adherence including poor medication efficacy [21, 22], adverse drug reactions [21, 23], unavailability of medications [24, 25], preference for complementary and alternative medicine [26, 27], spirituality [28], personality traits of patients [29], distance of hospital from home [30], displeasure with treatment [31], degree of insight the patient has into their illness [32], attitude to medication [33], and characteristics of the prescriber [34].

Therapeutic drug monitoring (TDM) is the measurement of concentrations of medications in blood to help measure adherence, individualize dose, observe the incidence of adverse effects, and determine how well patients respond to treatment [35, 36]. A few studies have been conducted in psychiatric settings to help establish the importance of TDM [37].

Studies have been conducted in various settings to examine the complex context of psychotropic medicine use [38]. Despite the emerging evidence that antipsychotics are among the commonly prescribed psychiatric medications in Ghana [39], there is limited evidence on the underlying factors and outcomes of adherence to these medications. The aim of this study was to address the research gap. We aimed to (1) identify the commonly prescribed psychotropic medications for patients with schizophrenia, (2) document the severity and nature of side effects experienced by these patients, (3) determine the rate of adherence and the causes of poor adherence among patients, (4) document the types of therapeutic monitoring employed in the psychiatric hospitals, and (5) investigate the relationship between the side effects experienced and medication adherence.

## 2. Methods

### 2.1. Study Design and Site

A descriptive hospital-based cross-sectional study was conducted at the Accra Psychiatric Hospital (APH) and Pantang Hospital (PH), the two government psychiatric hospitals located in the Greater Accra Region of Ghana. Data were collected between December 27, 2013, and January 30, 2014. The APH and PH offer clinical services such as counselling and therapy for admitted patients and outpatients, as well as training for practitioners.  Table 1 shows the distribution of staff at APH and PH.

In APH, the daily average of outpatients' attendance is 120 and an average of 5 patients are admitted. In PH, the daily average attendance of outpatients is 65 and an average of 4 are admitted.

### 2.2. Study Participants

The study participants included both inpatients and outpatients with schizophrenia (diagnosis in the clinical records) taking psychotropic drugs, aging 18 years and older, and having obtained a score of 23 and above on the Folstein Mini–Mental State Examination (MMSE) [40]. Willingness to participate also formed part of the inclusion criteria. However, patients exempted from the study were individuals with severe mental illnesses, patients who just commenced their treatment, those showing signs of aggression when asked to participate, and those who had not been prescribed psychotropic medications.

The minimum number of participants required for the study was determined using

*n* = *Z* ^2^ *p*(1 − *p*)/*d* ^2^, where *Z* is the level of confidence (1.96), *P* is the prevalence of patients being treated in the hospitals (0.104), and *d* is the precision (0.05) resulting in a minimum of 143 patients [41].

### 2.3. Study Instrument

Two types of assessment tools were administered to the participants: (1) the mental state assessment using the Folstein Mini–Mental State Examination (MMSE); (2) a questionnaire comprising questions in determining the stated objectives. The MMSE measures patients' orientation (total score of 10), registration (3), attention and calculation (5), recall (5), language (2), repetition (1), and complex commands (6). The MMSE is a 30-point test which indicates cognitive impairment when a score lower than 23 is obtained [40]. Since severely cognitively impaired patients with schizophrenia may not have the mental capacity to provide valid consent and responses for the study, participants scoring below the predetermined measure of 23 were excluded from the study.

The second questionnaire was made up of five sections: A–E. Section A gathered information on sociodemographic data, section B focused on the types of medications prescribed, Section C addressed issues of adherence and reasons for nonadherence, Section D obtained information on side effects of psychotropic medications used, and section E assessed therapeutic monitoring.

The 22- item Glasgow antipsychotic side effect scale (GASS) measured the types and extent of side effects of the psychotropic drugs experienced by patients [21]. The total GASS scores ranged from 0 to 63 and patients were considered to experience mild or absent side effects if they scored 0 to 12, moderate side effects (13–26), and severe side effects (27–63) [42].

The Medication Adherence Questionnaire was used to assess medication adherence behaviour [43]. The questions covered medication intake behaviour of patients on issues of forgetfulness, carelessness, and not taking the medicines because they subjectively experienced an improvement or deterioration in medical symptoms. The total score ranged from zero to four representing low (4), moderate (2-3), and high (0) adherence, respectively. Low and moderate categories comprised poor adherence [44]. An additional open-ended question on reasons for poor adherence was included in the questionnaire and the responses were documented.

Section E consisted of a set of questions that aimed to examine the types of therapeutic monitoring performed which comprised the measurement of plasma or serum levels and indicators for TDM such as the documentation of adherence and side effects [45]. The questions also explored the measurement of vital signs and functions of organs such as kidney, liver, and thyroid, as well as the measurement of blood glucose and drug levels in patients as indicated in the patient records. This helps to identify any heart, kidney, and liver dysfunctions likely to develop with long-term psychotropic medication use.

### 2.4. Statistical Analysis

The data were analyzed using Statistical Package for the Social Sciences (SPSS) version 21.0. Data were categorized and analyzed using both descriptive and inferential analysis including frequency distribution tables, Pearson correlation, and linear regression models.

### 2.5. Ethical Consideration

Ethical approval was obtained from each hospital before commencing fieldwork. Collection of data commenced after approval had been granted by both hospitals. Participation was voluntary, and informed consent was sought from all participants that enrolled for the study. Confidentiality was ensured by not indicating individuals' names or any identifiable information about participants.

## 3. Results

### 3.1. Sociodemographic Characteristics

Out of the 259 patients enrolled for the study, 218 were included in the analysis since they scored above the predetermined threshold of 23 out of the total score of 30 on the MMSE. However, the patient characteristics (*n* = 41) of those who scored lower than the set score have been presented in Tables 2 and 3. Most of them were males and on admission.

Table 4 shows the characteristics of the participants (*n* = 218) according to their sex, age, marital status, educational background, occupational status, and religious background. The majority of participants were males (61.9%), 18–29 years (29.4%), single (47.2%), having attained senior secondary education (36.2%), Christians (85.8%), and unemployed (43.1%). Termination of appointment (8.5%), experiences of side effects of medications (31.9%), cessation of formal education (13.8%), and stigma (45.7%) were the factors contributing to unemployment among the participants. The majority were inpatients (51.4%). However, the demographic profiles of participants who performed above the MMSE cut-off and those who performed below the cut-off did not differ. The MMSE was reliable in this study with Cronbach's alpha of 0.72.

### 3.2. Prescribed Psychotropic Drugs

Olanzapine was the commonly prescribed medication (Table 5). Patients were given trihexyphenidyl to help manage the negative muscarinic side effects associated with particularly the first-generation antipsychotic medications. No long acting antipsychotic injections were recorded. About 27.6% reported unavailability of their medicines.

### 3.3. Experiences of Side Effects

In Table 6, the majority of patients experienced side effects which were categorized as mild (73.4%) and moderate (26.6%). The negative effects were predominantly genitourinary (26%), gastrointestinal (17.2%), anticholinergic (13.9%), and cardiovascular (11.9%).

### 3.4. Monitoring of Side Effects

The major monitoring performed in the hospitals was the measurement of blood pressure and heart rate (33.6%) (Table 7). These were mostly done when medications were initiated. However, the frequency of these measurements declined after a period six of months. Therapeutic drug monitoring was rarely performed.

### 3.5. Adherence Behaviour

About 98% of the participants reported poor adherence levels to prescribed medications. As presented in Table 8, most of the participants indicated financial difficulties (14.6%) and forgetfulness (14.6%) as the main causes of nonadherence. No significant associations were observed for demographic characteristics, side effects, and adherence (Tables 9 and 10). The adherence measure was reliable in this study with Cronbach's alpha of 0.87.

## 4. Discussion

The main objective of the study was to find the commonly prescribed psychotropic medications from the perspective of patients with schizophrenia. Male patients were in the majority, and this is congruent with the fact that in 2011, out of 7993 admissions made to the three government mental hospitals in Ghana, 68% were males [46]. Similarly, the majority of participants were within the age range of 18–29 years, reflecting the evidence that the preponderance of Ghanaians suffering from mental disorders are young adults [47]. According to Lloyd [48], people with mental disorders have fewer job opportunities due to the stigmatization associated with the illness and the difficulty in keeping and finding a job. From the study, 43% of the respondents did not have any form of employment. Some patients lost their jobs when the mental illnesses began, others could not further their education, while others were stigmatized by their work colleagues. The Mental Health Act 846 in Ghana [2, 49] advocates that people living with mental illness should not be victimized or stigmatized and patients whose contracts are abrogated based on mental ill-health should be duly compensated as indicated in the Labour Act 651 of Ghana, yet, some patients had their contracts terminated without due compensation because they were considered unfit to carry out their duties.

Consistent with other studies, olanzapine was the most commonly prescribed psychotropic medication [50]. Atypical antipsychotics such as olanzapine are more frequently prescribed because they are less likely to cause extrapyramidal side effects as compared with the typical antipsychotics such as haloperidol [51, 52]. Trihexyphenidyl, which is an anticholinergic, was found to be frequently prescribed with olanzapine because it was used in managing the extrapyramidal effects associated with haloperidol. Haloperidol, a first-generation antipsychotic, has been found to produce highly pronounced extrapyramidal effects [53]. However, unavailability of these medicines was a major challenge for the patients, and this has been previously reported where access to psychiatric medicines is fraught by insufficient supply and cost challenges [39, 54]. In Ghana, since, by policy, treatment for mental disorders is not paid for at the government psychiatric hospitals, when medications run out, patients purchase the medicines privately [55]. In addition, no record of the use of long acting antipsychotic injections (LAIs) were found, and as has been reported in some studies, utilization of these LAIs is usually low due to factors such as cost, insurance coverage, treatment setting, negative attitudes of healthcare professionals and patients, fear, and pain on injection [33, 42].

According to Drummond [56], most of the side effects experienced with psychotropic drug use are usually bearable by patients. From the study, most patients experienced mild levels of side effects using the rating of the Glasgow antipsychotic side effects scale. The most common side effects experienced by these patients were related to the genitourinary system. The anticholinergic effects reported included blurred vision, dry mouth, and difficulty in passing urine which could progress to complications like increase in heart rate, tachyarrhythmias, urinary retention, and heart failure [57]. Cardiovascular side effects experienced with psychotropic drug use are common and some patients complained of this side effect [5]. According to Witchel et al. [58] and Narang et al. [59], users of psychotropic medications are twice more likely to have sudden cardiac death as compared with patients not on such medications. This effect can be attributed to the positive inotropic and chronotropic effects of the medicines, hence the need to monitor patients [60]. Similarly, the extrapyramidal symptoms (EPS) reported by patients showed various movement disorders such as acute dystonic reactions and pseudoparkinsonism which are usually managed with trihexyphenidyl and benztropine [61, 62]. Furthermore, the respondents experienced gastrointestinal side effects, particularly, constipation which can occur with psychotropic drug use [57]. Individuals on long-term antipsychotic medications such as olanzapine have a higher risk of developing diabetes compared with the general population and this confirmed the regular screen for blood glucose among the participants who often felt thirsty and passed urine frequently [63]. Further, the use antipsychotics could cause weight gain, which increases a patient's risk factor for other metabolic disorders such as hypertension, coronary heart disease, diabetes mellitus, and dyslipidaemias [64, 65]. Weight gain was reported by the study participants. A greater proportion of the patients had their vital signs monitored but the regularity of this practice decreased after 6 months. While blood pressure, heart rate, and blood glucose tests were primarily done at the psychiatric hospitals as part of patient monitoring, no assessments of medication concentrations in blood were performed as part of TDM for the antipsychotics particularly those with narrow therapeutic indices. This observation does not corroborate other studies on psychiatric patients [66–68]. It is essential to monitor schizophrenic patients taking psychotropic medications for better therapeutic outcome [69, 70].

In this study, 98% of patients poorly adhered to their medications because of economic challenges, forgetfulness, and cutting back on taking their medicines because they felt better or worse. Poor adherence has been reported from Nigeria (40.3%), Ethiopia (42.2%), and Pakistan (41.2%), and reasons such as distance from the hospital, low social support, and complex drug regimen were stated [31, 71].

The following study limitations are acknowledged. First, the study was limited to two public psychiatric hospitals. Therefore, the results obtained may not be generalizable to the experiences and views of patients from other public and private mental health facilities in Ghana. However, to the best of our knowledge, this study is the first to document the treatment regimes, side effects, types of therapeutic monitoring, and adherence behaviours among patients with schizophrenia in Ghana's two largest public psychiatric hospitals.

## 5. Conclusion

Adherence to medication in the Ghanaian psychiatric setting is a major public health problem. This occurs because of the economic challenges of patients, forgetfulness, and the feeling of wellness by the patients. To improve adherence and treatment outcomes for individuals with mental disorders, it is essential that mental health providers address these factors while providing appropriate support to improve medication adherence.

## Figures and Tables

**Table 1:** Staff distribution at Accra Psychiatric Hospital and Pantang Hospital.

Staff	APH	PH
Clinical psychologist	2	2
Psychiatrists	2	2
Medical officers	5	2
Nurses	435	329
Pharmacists	3	3
Physician medical assistants	7	-
Anaesthetics	3	-
Ward assistants	-	68
Biomedical scientists	0	2
Occupational therapists	0	8

**Table 2:** Sociodemographic data of study participants who performed below the cut-off for the MMSE.

Variable	Frequency	Percentage
*Sex*		
Male	23	56.1
Female	18	43.9
*Age*		
18–29	14	34.1
30–39	9	22.0
40–49	9	22.0
50–59	7	17.1
60–69	2	4.9
*Marital status*		
Single	19	46.3
Engaged	2	4.9
Married	10	24.4
Divorced	6	14.6
Separated	4	9.8
*Education*		
Nursery	1	2.4
Junior secondary school	11	26.8
Senior high school	19	46.3
Vocational	1	2.4
Polytechnic	1	2.4
Training school	2	4.9
University	6	14.6
*Religious affiliation*		
Atheist	3	7.3
Christian	35	85.4
Muslim	2	4.9
Traditionalist	1	2.4
*Occupational status*		
No occupation	16	39.0
Professor/technical	12	29.3
Managerial	1	2.4
Clerical	1	2.4
Sales	7	17.1
Agricultural self-employed	2	4.9
Household and domestic service	1	2.4
Skilled manual	1	2.4
*Reasons for unemployment*		
Termination of appointment	4	25.0
Side effects of medicines	6	37.5
Cessation of education	5	31.3
Stigma from coworkers	1	6.3
*Patient status*		
Inpatient	21	51.2
Outpatient	20	48.8

**Table 3:** Psychotropic drugs prescribed to patients who performed below the cut-off for the MMSE.

Psychotropic drug	Frequency	Percentage
Trihexyphenidyl	9	22.0
Fluoxetine	1	2.4
Carbamazepine	6	14.6
Haloperidol	8	19.5
Risperidone	5	12.2
Olanzapine	11	26.8
Sertraline	1	2.4

**Table 4:** Sociodemographic data of study participants who performed above the cut-off for the MMSE.

Variable	Frequency (218)	Percentage (100%)
*Sex*		
Male	135	61.9
Female	83	38.1
*Age*		
18–29	64	29.4
30–39	51	23.4
40–49	42	19.3
50–59	40	18.3
60–69	17	7.8
70–79	4	1.8
*Marital status*		
Single	103	47.2
Engaged	16	7.3
Married	62	28.4
Divorced	20	9.2
Separated	8	3.7
Living together	1	0.5
Widowed	8	3.7
*Education*		
Not applicable	4	1.8
Nursery	1	0.5
Primary	18	8.3
Junior secondary school	39	17.9
Senior high school	79	36.2
Vocational	8	3.7
Polytechnic	9	4.1
Training school	19	8.7
University	38	17.4
Masters	3	1.4
*Religious affiliation*		
Atheist	13	6.0
Christian	187	85.8
Muslim	15	6.9
Traditionalist	3	1.4
*Occupational status*		
No occupation	94	43.1
Professor/technical	38	17.4
Managerial	7	3.2
Clerical	4	1.8
Sales	41	18.8
Agricultural self-employed	7	3.2
Agriculture	5	2.3
Household and domestic service	13	6.0
Skilled manual	6	2.8
Unskilled manual	2	0.9
Others	1	0.5
*Reasons for unemployment*		
Termination of appointment	8	8.5
Side effects of medicines	30	31.9
Cessation of education	13	13.8
Stigma from coworkers	43	45.7
*Patient status*		
Inpatient	112	51.4
Outpatient	106	48.6

**Table 5:** Psychotropic drugs prescribed to patients who performed above the cut-off for the MMSE.

Psychotropic drug	Frequency	Percentage
Trihexyphenidyl	49	21.4
Fluoxetine	1	0.4
Carbamazepine	43	18.8
Haloperidol	36	15.7
Risperidone	30	13.1
Olanzapine	68	29.7
Sertraline	2	0.9

**Table 6:** Severity and types of side effects experienced by participants.

Variable	Frequency	Percentage
*Severity of side effects*		
Mild	160	73.4
Moderate	58	26.6
*Types of side effects experienced*		
Sedation and other CNS effects	44	5.4
Cardiovascular	97	11.9
Extrapyramidal	55	6.7
Anticholinergic	114	13.9
Gastrointestinal	141	17.2
Genitourinary	213	26.0
Frequent thirst with frequent urination	78	9.5
Weight gain	76	9.3

CNS: Central Nervous System. There are multiple responses to the types of side effects experienced.

**Table 7:** Monitoring of side effects.

Type of monitoring	Frequency	Percentage
*At initiation of psychotropic medication*		
Blood pressure & heart rate	226	33.6
Weight	157	23.3
Temperature	207	30.6
Fasting blood glucose	83	12.3
Lipids	1	0.1
*At least 6 months after drug initiation*		
Blood pressure & heart rate	159	32.8
Weight	135	27.8
Temperature	152	31.3
Fasting blood glucose	39	8.1
*As indicated in patient records*		
Blood pressure & heart rate	178	33.7
Weight	141	26.8
Temperature	170	32.2
Fasting blood glucose	37	6.9
Lipids	1	0.2
Complete blood count	1	0.2
Liver function	1	0.2

There are multiple responses to the type of therapeutic monitoring conducted. No measurements for drug levels in blood were conducted.

**Table 8:** Patient report of adherence to psychotropic medications.

Variable	Frequency	Percentage
*Adherence to psychotropic medication*		
Low	60	28.0
Moderate	150	70.1
High	4	1.9
*Reasons for poor adherence*		
Financial difficulties	23	14.6
Forgetfulness	23	14.6
Feeling of wellness	15	9.6
Disliking the chronic use of medicines	14	9.0
Busy schedule	14	9.0
Relapse of illness	7	4.5
Preference for CAM	5	3.4
Negative attitude of nurses	4	2.2
Distance to the hospital	4	2.2
Change in medication	4	2.2
Negative impact on career	2	1.1

Low and moderate adherence have been combined as poor adherence: 98.1%; CAM: complementary and alternative medicine.

**Table 9:** Relationship between demographic characteristics and adherence to psychotropic medications.

Variables	Medical adherence		*P* value
	Poor	High	
*Sex of respondent*			
Male	133 (62.1)	1 (25.0)	0.063
Female	81 (37.9)	3 (75.0)	
*Age of respondent*			2.968
18–39	114 (54.3)	2 (50.0)	
40–59	79 (37.6)	2 (50.0)	
60 and above	17 (8.1)	0 (0.0)	
*Marital status*			
Married	62 (29)	0 (0.0)	1.619
Not married	152 (71.0)	4 (100.0)	
*Educational level*			
No education	4 (1.9)	0 (0.0)	1.236
Low education	55 (25.7)	2 (50.0)	
High education	155 (72.4)	2 (50.0)	
*Occupation*			
No occupation	93 (43.5)	2 (50.0)	0.068
Working	121 (56.5)	2 (50.0)	
*Religion*			
Atheist	12 (5.6)	1 (33.3)	0.51
Christian	180 (84.1)	2 (66.7)	
Muslim	15 (7.0)	0 (0.0)	
Traditionalist	3 (1.4)	0 (0.0)	
*Patient status*			1.217
Inpatients	113 (52.8)	1 (25.0)	
Outpatients	101 (47.2)	3 (75.0)	

*P* < 0.01 is significant.

**Table 10:** Relationship between severity of side effect and adherence to psychotropic medications.

Variables	Medical adherence		*P* value
	Low	High	
*Severity of side effect*			
Mild	156 (72.9)	2 (50.0)	1.032
Moderate	58 (27.1)	2 (50.0)	

*P* < 0.01 is significant.

## References

[1] Dovi M. (2013). The silent crisis: Mental health in Africa. *Consultancy Africa Intelligence's Public Health Unit Discussion Paper*.

[2] Doku V. C., Wusu-Takyi A., Awakame J. (2012). Implementing the Mental Health Act in Ghana: any challenges ahead?. *Ghana Medical Journal*.

[3] World Health Organization (October 2007). *The country summary series*.

[4] Forster E. B. (2012). A historical survey of psychiatric practice in Ghana. *Ghana Medical Journal*.

[5] Read U. M., Doku V., de-Graft A., Aikins., Akyeampong E., Hill A., Kleinman A. (2015). Schizophrenia and Psychosis in West Africa from the colonial era to the present. *The Culture of Mental Illness and Psychiatric Practice in Africa*.

[6] Sikich L., Frazier J. A., McClellan J. (2008). Double-blind comparison of first- and second-generation antipsychotics in early-onset schizophrenia and schizoaffective disorder: Findings from the treatment of early-onset schizophrenia spectrum disorders (TEOSS) study. *The American Journal of Psychiatry*.

[7] Patel K. R., Cherian J., Gohil K., Atkinson D. (2014). Schizophrenia: Overview and treatment options. *Pharmacy and Therapeutics*.

[8] Eaton W. W., Chen C.-Y., Bromet E. J. (2011). Epidemiology of Schizophrenia. *Textbook in Psychiatric Epidemiology: Third Edition*.

[9] Bruijnzeel D., Suryadevara U., Tandon R. (2014). Antipsychotic treatment of schizophrenia: an update. *Asian Journal of Psychiatry*.

[10] Lage M. J., Hassan M. K. (2009). The relationship between antipsychotic medication adherence and patient outcomes among individuals diagnosed with bipolar disorder: a retrospective study. *Annals of General Psychiatry*.

[11] Osterberg L., Blaschke T. (2005). Adherence to medication. *The New England Journal of Medicine*.

[12] Gilmer T. P., Ojeda V. D., Barrio C. (2009). Adherence to antipsychotics among Latinos and Asians with schizophrenia and limited English proficiency. *Psychiatric Services*.

[13] Ascher-Svanum H., Zhu B., Faries D., Furiak N., Montgomery W. (2009). Medication adherence levels and differential use of mental-health services in the treatment of schizophrenia. *BMC Research Notes*.

[14] Kennedy S. H. (2006). A review of antidepressant treatments today. *European Neuropsychopharmacology*.

[15] Serretti A., Chiesa A., Calati R. (2013). Side effects associated with psychotropic medications in patients with bipolar disorder: Evidence from two independent samples. *Journal of Psychopharmacology*.

[16] Kazadi N. J. B., Moosa M. Y. H., Jeenah F. Y. (2008). Factors associated with relapse in schizophrenia. *South African Journal of Psychiatry*.

[17] Emsley R., Chiliza B., Asmal L., Harvey B. H. (2013). The nature of relapse in Schizophrenia. *BMC Psychiatry*.

[18] Kishimoto T., Agarwal V., Kishi T., Leucht S., Kane J. M., Correll C. U. (2013). Relapse prevention in schizophrenia: a systematic review and meta-analysis of second-generation antipsychotics versus first-generation antipsychotics. *Molecular Psychiatry*.

[19] Akpalu B., Lund C., Doku V. (2010). Scaling up community-based services and improving quality of care in the state psychiatric hospitals: The way forward for Ghana. *South African Journal of Psychiatry*.

[20] Xiao J., Mi W., Li L., Shi Y., Zhang H. (2015). High relapse rate and poor medication adherence in the chinese population with schizophrenia: Results from an observational survey in the people’s Republic of China. *Neuropsychiatric Disease and Treatment*.

[21] Higashi K., Medic G., Littlewood K. J., Diez T., Granström O., de Hert M. (2013). Medication adherence in schizophrenia: Factors influencing adherence and consequences of nonadherence, a systematic literature review. *Therapeutic Advances in Psychopharmacology*.

[22] Haddad P., Brain C., Scott J. (2014). Nonadherence with antipsychotic medication in schizophrenia: challenges and management strategies. *Patient Related Outcome Measures*.

[23] Birnbaum M., Sharif Z. (2008). Medication adherence in schizophrenia: Patient perspectives and the clinical utility of paliperidone ER. *Patient Preference and Adherence*.

[24] Endale Gurmu A., Abdela E., Allele B., Cheru E., Amogne B. (2014). Rate of nonadherence to antipsychotic medications and factors leading to nonadherence among psychiatric patients in gondar university hospital, Northwest Ethiopia. *Advances in Psychiatry*.

[25] Teferra S., Hanlon C., Beyero T., Jacobsson L., Shibre T. (2013). Perspectives on reasons for non-adherence to medication in persons with schizophrenia in Ethiopia: A qualitative study of patients, caregivers and health workers. *BMC Psychiatry*.

[26] Werneke U., Turner T., Priebe S. (2006). Complementary medicines in psychiatry: review of effectiveness and safety. *The British Journal of Psychiatry*.

[27] Qureshi N. A., Al-Bedah A. M. (2013). Mood disorders and complementary and alternative medicine: a literature review. *Neuropsychiatric Disease and Treatment*.

[28] Grover S., Davuluri T., Chakrabarti S. (2014). Religion, spirituality, and schizophrenia: a review. *Indian Journal of Psychological Medicine*.

[29] Schroeder K., Naber D., Huber C. G. (2014). Maladaptive personality traits increase subjectively during the course of schizophrenia spectrum disorders. *The Journal of Nervous and Mental Disease*.

[30] Kasckow KFelmet J., Appelt C., Thompson R., Rotondiand A., Haas G. (2013). Telepsychiatry in the assessment and treatment of schizophrenia. *Clinical schizophrenia related psychoses*.

[31] Noordraven E. L., Wierdsma A. I., Blanken P., Bloemendaal A. F. T., Mulder C. L. (2016). Depot-medication compliance for patients with psychotic disorders: The importance of illness insight and treatment motivation. *Neuropsychiatric Disease and Treatment*.

[32] Yalcin-Siedentopf N., Wartelsteiner F., Kaufmann A. (2015). Measuring adherence to medication in schizophrenia: the relationship between attitudes toward drug therapy and plasma levels of new-generation antipsychotics. *International Journal of Neuropsychopharmacology*.

[33] Kaplan G., Casoy J., Zummo J. (2013). Impact of long-acting injectable antipsychotics on medication adherence and clinical, functional, and economic outcomes of schizophrenia. *Patient Preference and Adherence*.

[34] Adelufosi A. O., Adebowale T. O., Abayomi O., Mosanya J. T. (2012). Medication adherence and quality of life among Nigerian outpatients with schizophrenia. *General Hospital Psychiatry*.

[35] Hales R. E., Yudofsky S. C., Roberts L. W. (2010). *The American Psychiatric Publishing Textbook of Psychiatry*.

[36] Loh G. W., Mabasa V. H., Ensom M. H. H. (2010). Therapeutic drug monitoring in the neurocritical care unit. *Current Opinion in Critical Care*.

[37] Conca A., Schmidt E., Pastore M., Hiemke C., Duffy D., Giupponi G. (2011). Therapeutic drug monitoring in Italian psychiatry. *Pharmacopsychiatry*.

[38] Maher A. R., Maglione M., Bagley S. (2011). Efficacy and comparative effectiveness of atypical antipsychotic medications for off-label uses in adults: a systematic review and meta-analysis. *Journal of the American Medical Association*.

[39] Oppong S., Kretchy I. A., Imbeah E. P., Afrane B. A. (2016). Managing mental illness in Ghana: The state of commonly prescribed psychotropic medicines. *International Journal of Mental Health Systems*.

[40] Folstein M. F., Folstein S. E., McHugh P. R. (1975). Mini-mental state: a practical method for grading the cognitive state of patients for the clinician. *Journal of Psychiatric Research*.

[41] Naing L., Winn T., Rusli B. N. (2006). Practical Issues in Calculating the Sample Size for Prevalence Studies. *Arch Orofac Sci*.

[42] Waddell L., Taylor M. (2008). A new self-rating scale for detecting atypical or second-generation antipsychotic side effects. *Journal of Psychopharmacology*.

[43] Morisky D. E., Green L. W., Levine D. M. (1986). Concurrent and predictive validity of a self-reported measure of medication adherence. *Medical Care*.

[44] Ross S., Walker A., MacLeod M. J. (2004). Patient compliance in hypertension: Role of illness perceptions and treatment beliefs. *Journal of Human Hypertension*.

[45] Hiemke C., Baumann P., Bergemann N. (2011). AGNP consensus guidelines for therapeutic drug monitoring in psychiatry: update 2011. *Pharmacopsychiatry*.

[46] Roberts M., Asare J., Mogan C., Adjase E., Osei A. (2013). *The mental health system in Ghana-WHO AIMS report*.

[47] Patel V., Leon D., Walt G. (2001). Poverty, inequality and mental health in developing countries. *Poverty, inequality and health*.

[48] Lloyd C. (2010). *Vocational rehabilitation and mental health*.

[49] Parliament of the Republic of Ghana Act 846 Mental Health Act. https://www.google.com/search?tbs=bks:1&amp;q=isbn:0724533613&amp;gws_rd=ssl#q=mental+health+act+846.

[50] Sarkar P., Chakraborty K., Misra A., Shukla R., Swain S. P. (2013). Pattern of psychotropic prescription in a tertiary care center: A critical analysis. *Indian Journal of Pharmacology*.

[51] Barlow D. H., Durand V. M. (2011). *Abnormal psychology: An integrative approach*.

[52] Culpepper L. (2007). A roadmap to key pharmacologic principles in using antipsychotics. *Primary Care Companion to the Journal of Clinical Psychiatry*.

[53] Brophy K. M., Scarlett-Ferguson H., Webber K. S., Abrams A. C., Lammon C. B. (2010). Clinical drug therapy for Canadian practice. *Lippincott Williams and Wilkins*.

[54] Raja S., Kippen S., Reich M. R., Akyeampong E., Hill A., Kleinman A. (2009). Access to psychiatric medicines in Africa. *Culture, Mental Illness and Psychiatric Practice in Africa*.

[55] Ofori-Atta A., Read U. M., Lund C. (2010). A situation analysis of mental health services and legislation in Ghana: Challenges for transformation. *South African Journal of Psychiatry*.

[56] Drummond E. H. (2006). *The Complete Guide to Psychiatric Drugs: Straight Talk for Best Results*.

[57] Mintzer J., Burns A. (2000). Anticholinergic side-effects of drugs in elderly people. *Journal of the Royal Society of Medicine*.

[58] Witchel H. J., Hancox J. C., Nutt D. J. (2003). Psychotropic drugs, cardiac arrhythmia, and sudden death. *Journal of Clinical Psychopharmacology*.

[59] Narang P., El-Refai M., Parlapalli R. (2010). Antipsychotic drugs: Sudden cardiac death among elderly patients. *Psychiatry*.

[60] Rosenberg D. R., Gershon S. (2002). *Pharmacotherapy for child and adolescent psychiatric disorders*.

[61] Kamin J., Manwani S., Hughes D. (2000). Extrapyramidal side effects in the psychiatric emergency service. *Psychiatric Services*.

[62] Virani A. S., Bezchlibnyk-butler K. Z., Jeffries J. J., Procyshyn R. M. (2012). *Clinical handbook of psychotropic drugs*.

[63] De Hert M., Detraux J., Van Winkel R., Yu W., Correll C. U. (2012). Metabolic and cardiovascular adverse effects associated with antipsychotic drugs. *Nature Reviews Endocrinology*.

[64] Shaw J., DeMaso D. R. (2007). *Clinical manual of pediatric psychosomatic medicine: Mental health consultation with physically ill children and adolescents*.

[65] Ruetsch O., Viala A., Bardou H., Martin P., Vacheron M. N. (2005). Psychotropic drugs induced weight gain: A review of the literature concerning epidemiological data, mechanisms and management. *L'Encéphale*.

[66] White W., Elmore L., Luthin D. R., Cates M. E. (2013). Psychotropic-induced weight gain: a review of management strategies. *Consultant*.

[67] Guo W., Guo G.-X., Sun C. (2013). Therapeutic drug monitoring of psychotropic drugs in china: a nationwide survey. *Therapeutic Drug Monitoring*.

[68] Gex-Fabry M., Balant-Gorgia A. E., Balant L. P. (2003). Therapeutic drug monitoring of olanzapine: The combined effect of age, gender, smoking, and comedication. *Therapeutic Drug Monitoring*.

[69] Schoretsanitis G., Spina E., Hiemke C., de Leon J. (2017). A systematic review and combined analysis of therapeutic drug monitoring studies for long-acting risperidone. *Expert Review of Clinical Pharmacology*.

[70] Ramankutty G. (2002). Olanzapine-induced destabilization of diabetes in the absence of weight gain. *Acta Psychiatrica Scandinavica*.

[71] Lambert T. (2011). Managing the metabolic adverse effects of antipsychotic drugs in patients with psychosis. *Australian Prescriber*.

